# Epigenetics, N-myrystoyltransferase-1 and casein kinase-2-alpha modulates the increased replication of HIV-1 CRF02_AG, compared to subtype-B viruses

**DOI:** 10.1038/s41598-019-47069-9

**Published:** 2019-07-23

**Authors:** Biju Bhargavan, Georgette D. Kanmogne

**Affiliations:** Department of Pharmacology and Experimental Neuroscience, University of Nebraska Medical Center, Omaha, Nebraska 68198-5800 USA

**Keywords:** Transcription, HIV infections

## Abstract

HIV subtypes distribution varies by geographic regions; this is likely associated with differences in viral fitness but the predictors and underlying mechanisms are unknown. Using *in-vitro*, *in-vivo*, and *ex-vivo* approaches, we found significantly higher transactivation and replication of HIV-1-CRF02_AG (prevalent throughout West-Central Africa), compared to subtype-B. While CRF02_AG-infected animals showed higher viremia, subtype-B-infected animals showed significantly more weight loss, lower CD4+ T-cells and lower CD4/CD8 ratios, suggesting that factors other than viremia contribute to immunosuppression and wasting syndrome in HIV/AIDS. Compared to HIV-1-subtype-B and its Tat proteins(Tat.B), HIV-1-CRF02_AG and Tat.AG significantly increased histone acetyl-transferase activity and promoter histones H3 and H4 acetylation. Silencing N-myrystoyltransferase(NMT)-1 and casein-kinase-(CK)-II-alpha prevented Tat.AG- and HIV-1-CRF02_AG-mediated viral transactivation and replication, but not Tat.B- or HIV-1-subtype-B-mediated effects. Tat.AG and HIV-1-CRF02_AG induced the expression of NMT-1 and CKII-alpha in human monocytes and macrophages, but Tat.B and HIV-1-subtype-B had no effect. These data demonstrate that NMT1, CKII-alpha, histone acetylation and histone acetyl-transferase modulate the increased replication of HIV-1-CRF02_AG. These novel findings demonstrate that HIV genotype influence viral replication and provide insights into the molecular mechanisms of differential HIV-1 replication. These studies underline the importance of considering the influence of viral genotypes in HIV/AIDS epidemiology, replication, and eradication strategies.

## Introduction

The human immunodeficiency virus (HIV) is characterized by a very high genetic variability, due to lack of DNA proofreading activity of the reverse transcriptase enzyme and pharmacological selective pressure^1–4^. This has resulted in mutations, high rates of intra- and inter-molecular recombination within infected hosts, and very high clade diversity^5–7^. Over 95% of people living with HIV/AIDS are infected with HIV-1^8,9^. HIV-1 includes four groups: M (major), O (outlier), N (non-M non-O), and P^1,5^. HIV-1 group M accounts for the vast majority of infection globally and includes 9 pure subtypes (A-D, F-H, J and K), sub-subtypes (A1 and A2, F1 and F2), about 96 circulating recombinant forms (CRFs) and several unique (unclassified) recombinant forms (URFs)^1,7^. HIV-1 CRF02_AG is a recombinant of subtypes A and G, circulating in West and Central Africa; with 52–84% of HIV-infected humans in that region infected with HIV-1 CRF02_AG^10–13^. This Central and West African region includes 26 countries with over 535 million inhabitants^14^.

HIV-1 subtype distribution varies by geographic regions, with subtype-B prevalent in north America, Europe and other Western countries, subtype-C prevalent in Eastern and Southern Africa and Asia, subtype-D prevalent in Eastern and Central Africa, while subtypes A, G, and CRF02_AG are prevalent in West and Central Africa^5–7^. This differential geographic distribution of HIV clades is likely associated with differences in viral fitness, replication capacity, and viral adaptation in a given environment. In fact, the transactivator of transcription (Tat) binds to the trans-activating response element (TAR) to modulate the expression of viral genes and HIV replication; and genetic variations in the Tat region has been shown to affects its interaction with TAR and viral replication^15,16^. The host genetics, ethnicity and immune response also drive HIV genetic changes and play a major role in the control of viral replication, mutations, immune response, and disease progression^17,18^.

Viral genotype can influence Tat-induced blood-brain barrier inflammation^19,20^, cytokine expression and chemotactic activities^20,21^, and can affect the progression to AIDS^22,23^. *Ex-vivo* and *in-vitro* studies also showed increased replication of HIV-1 CRF02_AG isolates compared to subtypes A and G viral isolates^24,25^. The molecular mechanisms modulating this subtype-based differential viral replication are not known. In the present study, using cell lines, primary HIV-1 isolates, human peripheral blood mononuclear cells (PBMC), monocytes-derived macrophages (MDM), and HIV/AIDS animal models, we show significantly higher replication of primary HIV-1 CRF02_AG (AG) isolates *in-vitro* and *in-vivo*, compared to subtype-B viral isolates. Mechanistic studies demonstrate that this increased replication of AG isolates was associated with increased histone acetyl transferase (HAT) activity, increased acetylation of histone H3 and H4 in the viral long-terminal repeat (LTR) region, increased viral promoter transactivation by Tat proteins derived from HIV-1 CRF02_AG (Tat.AG) and primary CRF02_AG viral isolates (but not Tat.B or HIV-1 subtype-B). We demonstrate that Tat.AG and HIV-1 CRF02_AG (but not Tat.B or HIV-1 subtype-B) increased N-myrystoyltransferase (NMT)-1 and casein kinase-II-alpha (CKIIα) expression in human monocytes and MDM, and silencing NMT1 and CKIIα (but not NMT2 or CKIIβ) genes blocked HIV-1 CRF02_AG (but not subtype B) infection of human macrophages. These mechanistic studies provide insights into the molecular mechanisms modulating the increased replication of CRF02_AG viruses, and have implications for the transmission, adaptation, and predominance of this recombinant virus in Sub-Saharan Africa.

## Results

### Increased replication of primary HIV-1 CRF02_AG isolates, compared to HIV-1 subtype-B, in human blood cells

**Figure 1:**
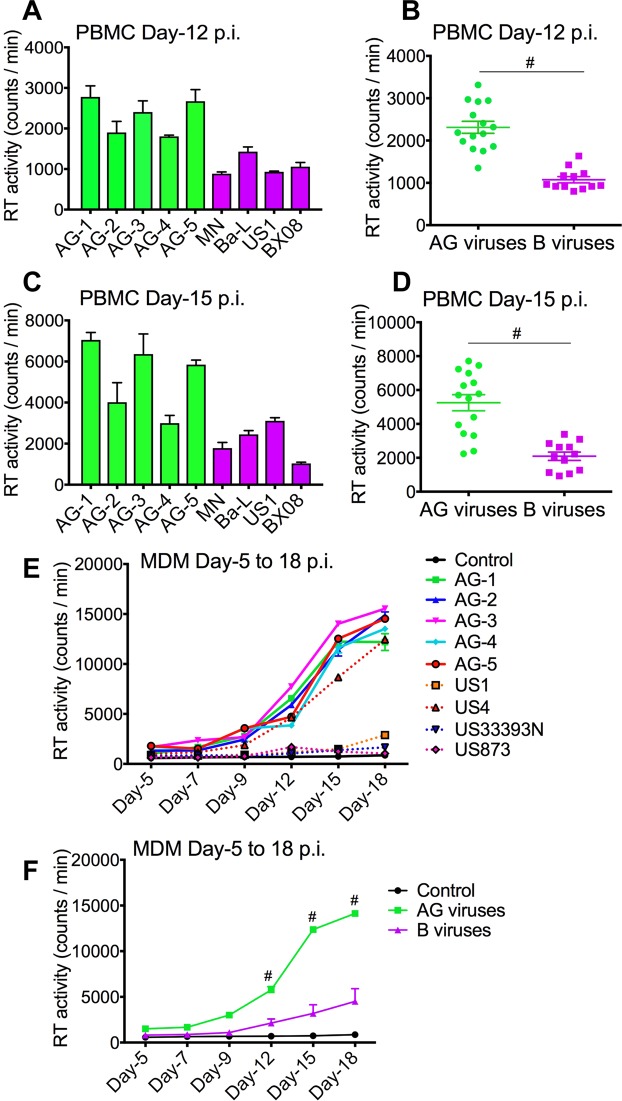
Increased replication of primary HIV-1 CRF02_AG isolates in human PBMC and MDM, compared to clade-B viruses. Reverse transcriptase activity in primary human PBMC (**A**–**D**) and MDM (**E**,**F**) infected with AG and clade-B viruses. RT activity assessed at day-12 (**A**,**B**) and day-15 (**C**,**D**) p.i. in PBMC, and from day-5 to day-18 p.i. in MDM (**E**,**F**). ^#^P < 0.0001. Errors bars represent SEM.

HIV replicative capacity determines its fitness and adaptation in a given environment. Comparative analyses of the replication capacity of HIV-1 subtype-B (4 different isolates) and CRF02_AG (5 different isolates) in human PBMC and MDM showed that compared to PBMC infected with HIV-1 subtype-B, CRF02_AG-infected cells had significantly higher reverse transcriptase (RT) activity at day-12 (Fig. 1A,B) and day-15 (Fig. 1C,D) post-infection (p.i.) (P < 0.0001). Similarly, higher RT activity was seen in CRF02_AG-infected MDM from day-5 to day-18 p.i. compared to clade-B-infected MDM (Fig. 1E,F; P < 0.0001).

### Infection of NOD/*scid*–IL-2Rγ_c_^*null*^ (NSG) mice with HIV-1 subtype-B resulted in higher weight loss, compared to animals infected with HIV-1 CRF02_AG

**Figure 2:**
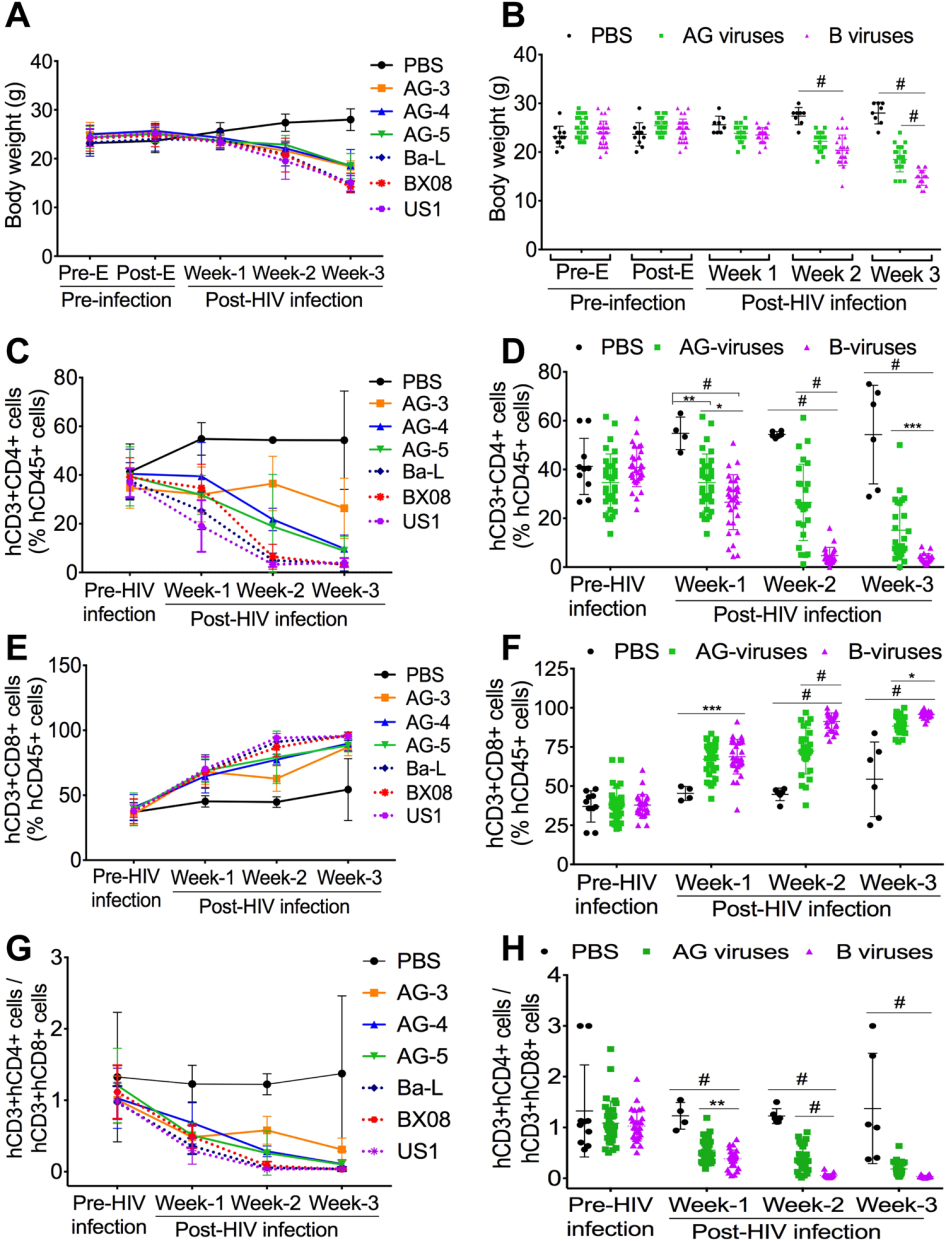
Increased immune suppression in animals infected with HIV-1 clade-B compared to CRF02_AG. **(A**,**B**) Body weight of control (PBS), HIV-1 CRF02_AG (AG), and clade-B (**B**) infected animals before (Pre-E) and after (Post-E) engraftment, before and after HIV-1 infection. (**C**–**H**) levels of human (h)CD4+ (**C**,**D**) and hCD8+ (**E**,**F**) T-cells in the blood of control, AG- and clade-B-infected animals pre-infection and at week-1, -2, and -3 post-infection; and hCD4/hCD8 ratios (**G**,**H**). *P < 0.05, **P < 0.01, ***P < 0.001, ^#^P < 0.0001. For all panels, errors bars represent SD.

To validate our *in-vitro* findings, we performed *in-vivo* experiments using NSG mice engrafted with human peripheral blood lymphocytes (PBL). Animals were monitored and weighed every 2–3 days before (pre-E) and after (post-E) engraftment, before and after infection. All engrafted animals (mock-infected controls, AG-infected, and clade-B-infected mice) had similar body weight pre- and post-engraftment and at week-1 p.i., mean mouse weight: 23–25.6 grams(g) (Fig. 2A,B). At week-2 p.i., compared to controls (mean weight 27.38 ± 1.77(SD) g/mouse), the weight of clade-B (20.38 ± 3.14 g/mouse) and AG (22.22 ± 1.74 g/mouse) infected mice were significantly lower (P < 0.0001, Fig. 2A,B), but there was no significant difference between AG- and clade-B-infected animals’ weight. At week-3 p.i., compared to controls (mean weight 28 ± 2.2 g/mouse), the weight of clade-B (14.73 ± 1.5 g/mouse) and AG (18.5 ± 2.55 g/mouse) infected mice were significantly lower (P < 0.0001); and the weight of clade-B infected mice was significantly lower than that of AG-infected animals (P < 0.0001, Fig. 2A,B).

### Infection of animals with HIV-1 subtype-B resulted in increased loss of CD4+ T-cells, compared to animals infected with HIV-1 CRF02_AG

The effect of HIV-1 infection on mice immune system was monitored pre- and post-infection by fluorescence activated cell sorting (FACS) quantification of human (hCD45+, hCD3+, hCD4+, and hCD8+) cells in animals’ blood. At week-1 post-E, all engrafted animals had similar levels of hCD4+ T-cells (37.8 ± 7 to 41 ± 11%) (mean ± SD) (Fig. 2C,D). Levels of hCD4+ T-cells gradually declined following HIV-1 infection, but this decrease was significantly larger in clade-B-infected mice compared to AG-infected animals. At week-1 p.i., hCD4+ T-cells levels in controls was 1.58-fold higher than in AG-infected animals (P = 0.0037, Fig. 2D), 2.06-fold higher than in clade-B-infected animals (P < 0.0001, Fig. 2D), and hCD4+ T-cells levels in AG-infected animals was 1.3-fold higher than in clade-B-infected animals (P = 0.018, Fig. 2D). At week-2 p.i., hCD4+ T-cells levels in controls was 2-fold higher than in AG-infected animals, 11.4-fold higher than in clade-B-infected animals, and hCD4+ T-cells levels in AG-infected animals was 5.6-fold higher than in clade-B-infected animals (P < 0.0001, Fig. 2D). At week-3 p.i., hCD4+ T-cells levels in non-infected controls was 4.45-fold higher than in AG-infected animals (P < 0.0001, Fig. 2D), 15-fold higher than in clade-B-infected animals (P < 0.0001, Fig. 2D), and hCD4+ T-cells levels in AG-infected animals was 4.36-fold higher than in clade-B-infected animals (P = 0.0006, Fig. 2D).

### Infection of animals with HIV-1 subtype-B resulted in higher hCD8+ T-cells and lower hCD4/hCD8 ratios, compared to animals infected with HIV-1 CRF02_AG

At week-1 post-E, all engrafted animals had similar levels of hCD8+ T-cells (37 ± 10 to 38.4 ± 10.4%) (mean ± SD) (Fig. 2E,F). hCD8+ T-cells levels gradually increased following infection, with larger increases in clade-B-infected mice, compared to AG-infected animals (Fig. 2E,F). At week-1 p.i., hCD8+ T-cells levels in AG- and clade-B-infected animals were respectively 1.48-fold (P = 0.0008, Fig. 2F) and 1.52-fold (P = 0.0003, Fig. 2F) higher than hCD8+ T-cells levels in control animals. At week-2 p.i., hCD8+ T-cells levels in AG- and clade-B-infected animals were respectively 1.6-fold and 2-fold higher than levels in control animals, and hCD8+ T-cells levels in clade-B-infected animals were 1.25-fold higher than in AG-infected animals (P < 0.0001, Fig. 2F). At week-3 p.i., hCD8+ T-cells levels in AG- and clade-B-infected animals were respectively 1.6-fold and 1.76-fold higher than levels in control animals, and levels in clade-B-infected animals were 7.7% higher than in AG-infected animals (P = 0.013, Fig. 2F).

Pre-infection ratios of hCD3 + hCD4 + /hCD3 + hCD8+ T-cells were similar for controls, AG-infected, and clade-B-infected animals (Fig. 2G,H). These ratios gradually decreased following HIV-1 infection. At week-1 p.i., hCD4/hCD8 ratios in AG- and clade-B-infected animals were respectively 2.22-fold and 3.2-fold lower than in control animals (Fig. 2G,H, P < 0.0001), and ratios in clade-B-infected animals were 1.44-fold lower than in AG-infected animals (Fig. 2G,H, P < 0.01). At week-2 p.i., hCD4/hCD8 ratios in AG- and clade-B-infected animals were respectively 3.12-fold and 22.27-fold lower than in control animals, and ratios in clade-B-infected animals were 7-fold lower than in AG-infected animals (Fig. 2G,H, P < 0.0001). At week-3 p.i., hCD4/hCD8 ratios in AG- and clade-B-infected animals were respectively 7.7-fold and 35.6-fold lower than in control animals (Fig. 2G,H, P < 0.0001); ratios in clade-B-infected animals were 4.6-fold lower than in AG-infected animals.

### Increased viremia in HIV-1 CRF02_AG-infected mice compared to clade-B-infected animals

**Figure 3:**
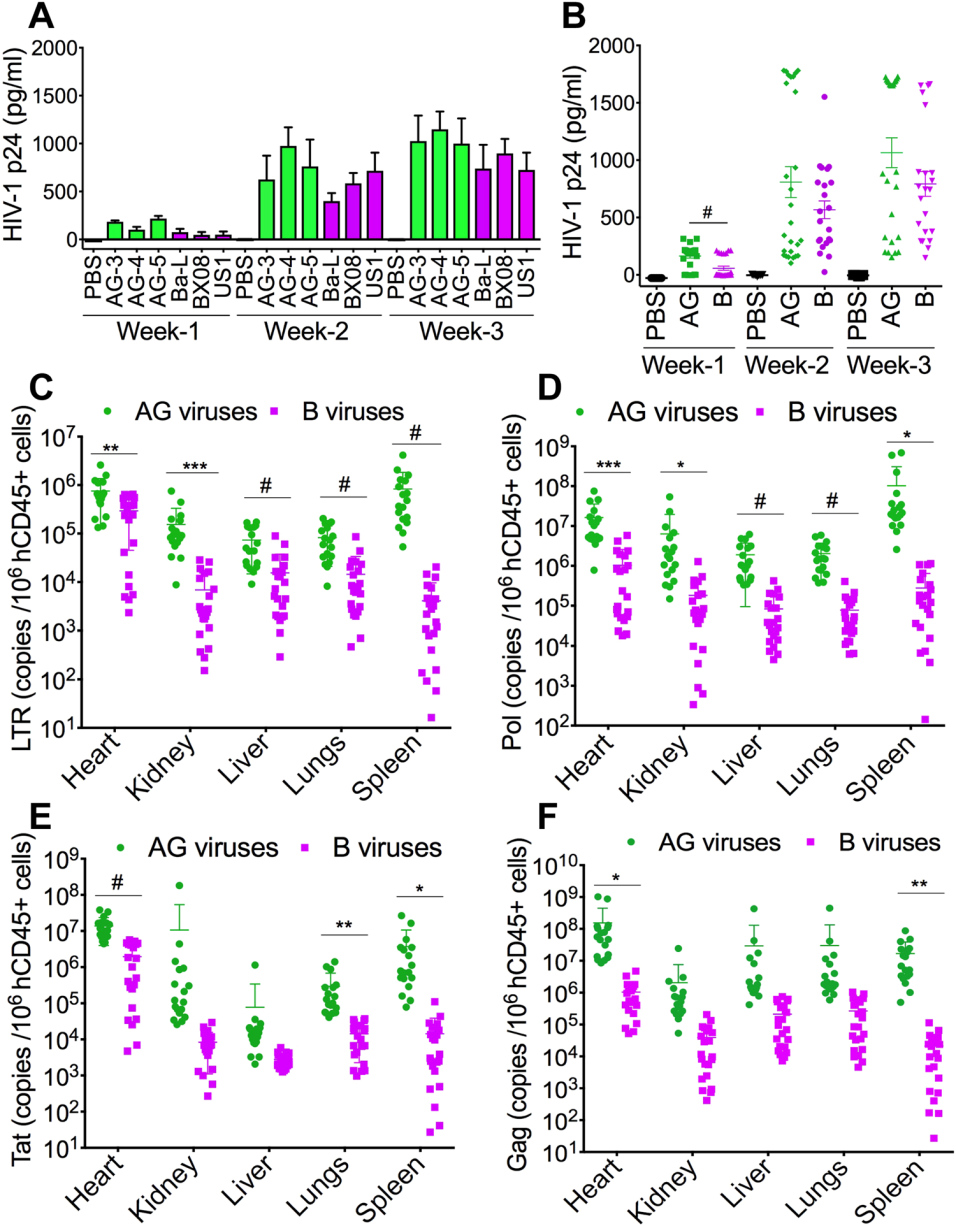
Increased viremia in the blood and tissues of animals infected with HIV-1 CRF02_AG (AG) compared to clade-B-infected animals. (**A**,**B**) Plasma HIV-1 p24 levels at week-1, -2, and -3 p.i. (**C**–**F**) quantitative PCR showing copies number of HIV-1 LTR (**C**), pol (**D**), tat (**E**), and gag (**F**) in animal’s heart, kidney, liver, lungs, and spleen. *P < 0.05, **P < 0.01, ***P < 0.001, ^#^P < 0.0001. For all panels, errors bars represent SD.

Studies in NSG mice, a well characterized HIV/AIDS animal model^26–28^, showed higher HIV-1 p24 antigen levels in plasma of AG-infected mice (N = 29) compared to clade-B infected mice (N = 25) (Fig. 3). At week-1 p.i., HIV-1 p24 antigen levels in AG-infected animals [164.2 ± 17 pg/ml (mean ± SD)] was 2.86-fold higher than p24 levels in clade-B-infected animals (57.37 ± 18.2 pg/ml) (Fig. 3A,B, P < 0.0001). At week-2 p.i., p24 levels in AG-infected animals (809 ± 134 pg/ml) was 1.4-fold higher than p24 levels in clade-B-infected animals (568 ± 78 pg/ml). At week-3 p.i., p24 levels in AG-infected animals (1065 ± 130.3 pg/ml) was 1.34-fold higher than in clade-B-infected animals (792.3 ± 108.2 pg/ml) (Fig. 3A,B). However, differences in week-2 and week-3 p24 levels did not reach statistical significance due to large variations between animals: at week-2 p.i., plasma p24 levels varied from 103.2–1781 (interquartile range (IQR): 172, 1726) pg/ml in AG-infected mice, and from 24.87–1551 (IQR: 292, 835.8) pg/ml in clade-B-infected mice. At week-3 p.i., plasma p24 levels varied from 154.2–1729 (IQR: 317, 1686) pg/ml in AG-infected mice, and from 148–1662 (IQR: 353, 1043) pg/ml in clade-B-infected mice.

### Increased viral loads in tissues of HIV-1 CRF02_AG-infected mice, compared to animals infected clade-B viruses

We performed real-time PCR quantification of HIV-1 LTR, pol, tat, and gag mRNA in animals’ tissues, including the heart, kidney, liver, lungs, and spleen. Data normalized to hCD45+ cells levels in tissue found that compared to clade-B-infected animals, LTR levels in AG-infected animals were significantly higher in the heart (2.56-fold, P = 0.0027), kidney (22.32-fold, P = 0.0005), liver (4.77-fold, P = 0.0001), lungs (5.7-fold, P < 0.0001), and spleen (200-fold, P = 0.0004) (Fig. 3C). AG-infected animals showed significantly higher levels of HIV-1 pol in the heart (15.73-fold, P = 0.0007), kidney (34.8-fold, P = 0.033), liver (22.85-fold, P < 0.0001), lungs (26-fold, P < 0.0001), and spleen (368-fold, P = 0.023) (Fig. 3D). AG-infected animals showed higher levels of HIV-1 tat in the heart (7.1-fold, P < 0.0001), kidney (1269-fold), liver (27.46-fold), lungs (20.3-fold, P = 0.002), and spleen (258.5-fold, P = 0.016) (Fig. 3E). Compared to clade-B-infected animals, AG-infected animals also showed higher levels of HIV-1 gag in the heart (147.3-fold, P = 0.019), kidney (52.5-fold), liver (137.5-fold), lungs (114.5-fold), and spleen (792.3-fold, P = 0.001) (Fig. 3F).

**Figure 4:**
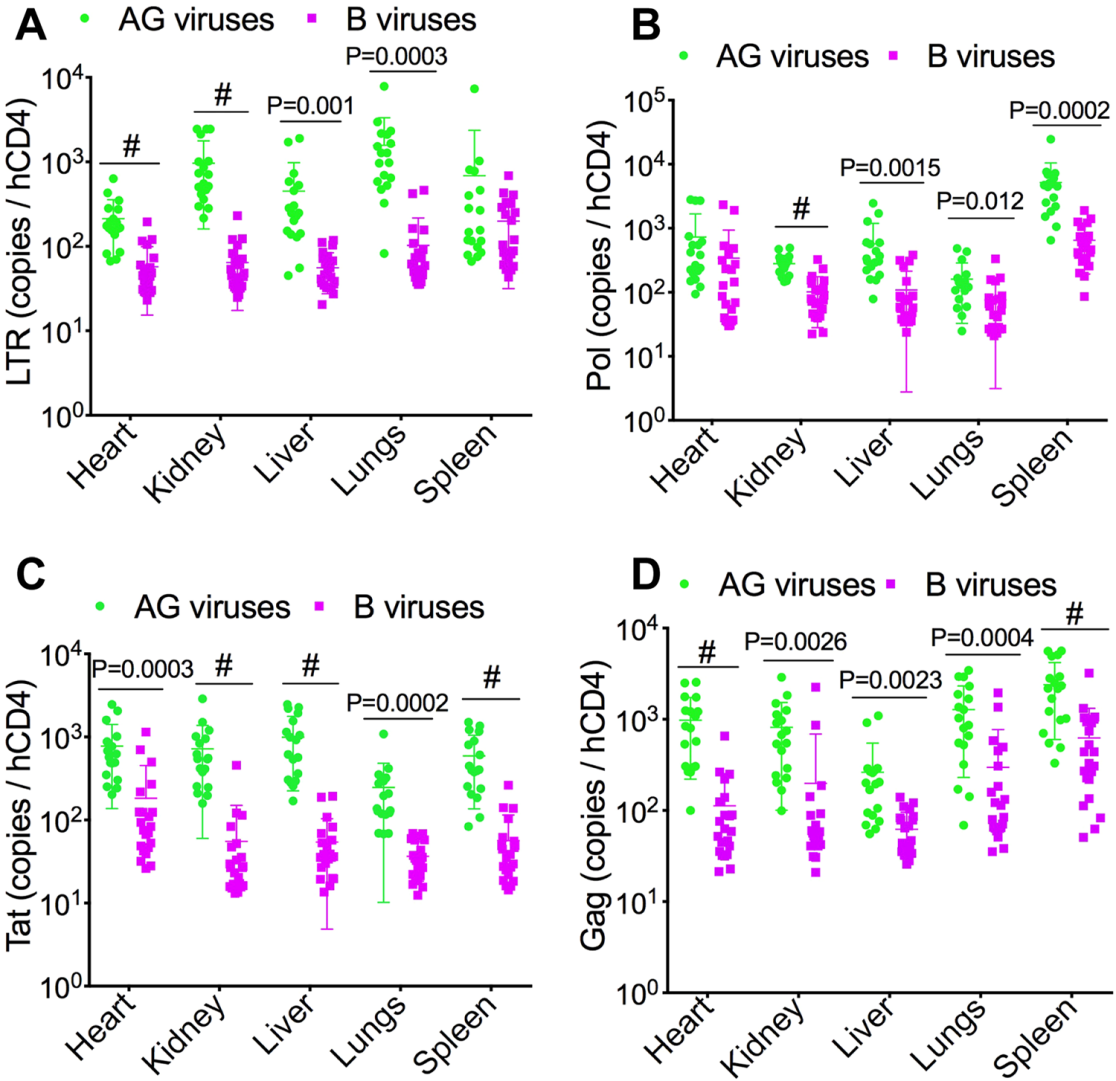
Data normalized to tissues hCD4 levels confirmed increased viremia in tissues of animals infected with HIV-1 CRF02_AG (AG) compared to clade-B-infected animals. (**A**–**D**) quantitative PCR showing copies number of HIV-1 LTR (**A**), pol (**B**), tat (**E**), and gag (**D**) in animal’s heart, kidney, liver, lungs, and spleen. *P < 0.05, **P < 0.01, ***P < 0.001, ^#^P < 0.0001. For all panels, errors bars represent SD.

Additional analyses normalized to hCD4+ cells levels in tissue found significantly increased viremia in AG-infected animals. Compared to clade-B-infected animals, HIV-1 LTR levels in AG-infected animals were significantly higher in the heart (3.7-fold, P < 0.0001), kidney (15-fold, P < 0.0001), liver (8-fold, P = 0.001), lungs (15.37-fold, P = 0.0003), and spleen 3.46-fold (Fig. 4A). AG-infected animals also showed higher HIV-1 pol levels in the heart (2.1-fold), kidney (2.76-fold, P < 0.0001), liver (5.3-fold, P = 0.0015), lungs (2.1-fold, P = 0.012), and spleen (8-fold, P = 0.0002) (Fig. 4B). AG-infected animals showed significantly higher levels of HIV-1 tat in the heart (4.25-fold, P = 0.0003), kidney (12.95-fold, P < 0.0001), liver (18.32-fold, P < 0.0001), lungs (6.74-fold, P = 0.0002), and spleen (10.5-fold, P < 0.0001) (Fig. 4C). Compared to clade-B-infected animals, AG-infected animals also showed significantly higher levels of HIV-1 gag in the heart (8.68-fold, P < 0.0001), kidney (4.1-fold, P = 0.0026), liver (4.2-fold, P = 0.0023), lungs (4.3-fold, P = 0.0004), and spleen (3.85-fold, P < 0.0001) (Fig. 4D).

### Increased LTR transactivation with Tat.AG and HIV-1 CRF02_AG compared to Tat.B and HIV-1 clade-B

**Figure 5:**
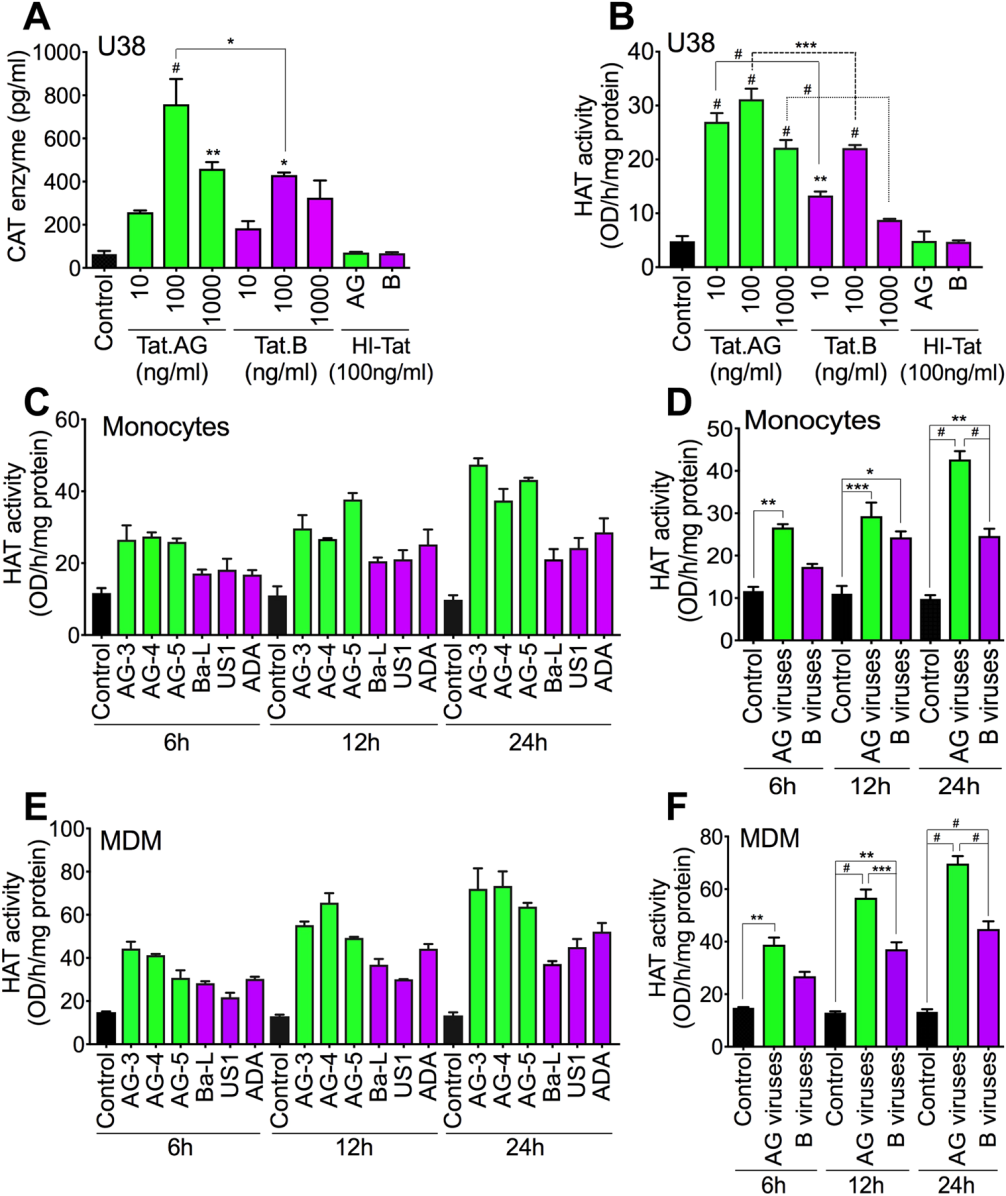
Tat.AG and HIV-1 CRF02_AG isolates induced higher LTR transactivation and increased HAT activity compared to Tat.B and HIV-1 clade-B. (**A**,**B)** Levels of CAT enzyme (**A**) and HAT activity (**B**) in U38 cells treated for 48 h with Tat.AG or Tat.B (10 to 1000 ng/ml). HI-Tat: heat-inactivated Tat proteins. Comparative p-values, or p-values compared to cells treated with HI-Tat proteins are shown. (**C**–**F)** HAT activity in primary human monocytes (**C**,**D**) and MDM (**E**,**F**) exposed to HIV-1 CRF02_AG or clade-B for 6 to 24 h. *P < 0.05, **P < 0.01, ***P < 0.001, ^#^P < 0.0001. (**A**–**C**,**E**) errors bars represent SD. (**D**,**F**) errors bars represent SEM.

Efficient and increased HIV-1 promoter activity correlate with increased LTR transcription and viral replication, and this is modulated by Tat binding to TAR^15,16^. Therefore, using U38 cells, we assessed the effects of Tat.AG and Tat.B on LTR promoter activity. Both Tat.AG and Tat.B (10–1000 ng/ml) significantly increased LTR transcriptional activity compared to cells exposed to heat-inactivated (HI) Tat proteins (Fig. 5A). LTR transcriptional activity in cells exposed to Tat.AG was 1.4–1.76-fold higher than LTR activity in cells exposed to Tat.B (P < 0.05, Fig. 5A).

### Increased viral transactivation with Tat.AG and HIV-1 CRF02_AG is associated with increased HAT activity, compared to Tat.B and HIV-1 clade-B

Because increased HAT activity is known to enhance HIV replication, we assessed the effects of Tat.AG and Tat.B on HAT activity in U38 cells. Both Tat.AG and Tat.B significantly increased HAT activity in U38 cells compared to cells exposed to heat-inactivated (HI)-Tat (Fig. 5B). HAT activity in cells exposed to Tat.AG was 1.4–2.53-fold higher than HAT activity in cells exposed to Tat.B (Fig. 5B). Because macrophages are some of the major HIV cellular reservoirs in humans, we assessed the effect of CRF02_AG and clade-B HIV-1 (3 different isolates for each subtype) on HAT activity in primary human monocytes and MDM. Compared to controls, exposure of human monocytes to AG isolates increased HAT activity by 2.66–4.34-fold whereas exposure of monocytes to clade-B HIV-1 isolates increased HAT activity by 1.5–2.5-fold (Fig. 5C,D); HAT activity in monocytes exposed to HIV-1 CRF02_AG isolates was 1.2–1.73-fold higher than HAT activity in monocytes exposed to clade-B isolates (Fig. 5C,D). Compared to non-infected MDM, exposure of human MDM to HIV-1 CRF02_AG isolates increased HAT activity by 2.6–5.24-fold whereas exposure of MDM to clade-B isolates increased HAT activity by 1.8–3.37-fold (Fig. 5E,F); HAT activity in MDM exposed to HIV-1 CRF02_AG isolates was 1.45–1.55-fold higher than HAT activity in MDM exposed to clade-B isolates (Fig. 5E,F).

### Increased HIV-1 transactivation and HAT activity with Tat.AG are associated with increased acetylation of histone H3 and H4 in the LTR promoter

**Figure 6:**
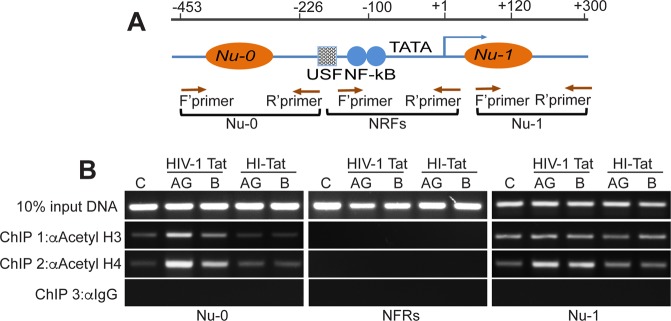
Increased acetylation of histone H3 and H4 by Tat.AG compared to Tat.B. (**A**) Schematic structure of the integrated HIV-1 LTR promoter; the position of nucleosomes (Nu)-0, Nu-1, and the nucleosome-free regions (NFRs) are shown, as well as the position of transcriptional elements known to control HIV transactivation. The blue arrow indicates the transcription start site. Brown arrows indicate the positions of the forward (F’) and reverse (R’) primers used for PCR amplification. (**B**) Quantification of acetylated H3 and H4 by PCR of crosslinked DNA in U38 cells, following 48 h treatment with Tat.AG, Tat.B or HI-Tat (all at 100 ng/ml), and ChIP with anti-acetylated histone H3, anti-acetylated histone H4, or control IgG antibodies.

Acetylation of histone proteins correlate with increased HAT activity, and HIV-1 transactivation is often associated with increased histone acetylation^29,30^. We performed Chromatin Immunoprecipitation (ChIP) assays to determine whether Tat.AG and Tat.B differentially induce acetylation of histones in the HIV-1 LTR promoter region(s) (Fig. 6A). Tat increased acetyl-H3 and acetyl-H4 levels in nucleosome (Nu)-0 and Nu-1 regions of the HIV-1 promoter. Compared to Tat.B, Tat.AG induced higher levels of acetyl-H3 and acetyl-H4 in Nu-0 (Fig. 6B). No acetylated histone was detected in the nucleosome-free regions (NFRs). Acetyl-H3 and acetyl-H4 levels in cells treated with similar concentrations of HI-Tat proteins were similar to levels in controls (C, Fig. 6B).

### NMT1 and CKIIα are involved in Tat.AG but not Tat.B mediated HIV-1 transactivation

**Figure 7:**
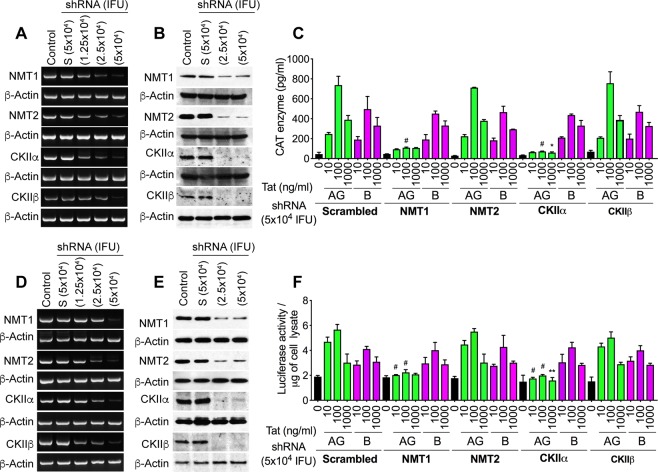
Silencing the NMT1 or CKIIα genes blocked Tat.AG but not Tat.B-induced HIV-1 transactivation in U38 (**A**–**C**) and TZM-bl (**D**–**F**) cells. (**A**,**D**) RT-PCR confirmation of NMT1, NMT2, CKIIα and CKIIβ genes silencing. (**B**,**E**) Western blot confirmation of NMT1, NMT2, CKIIα and CKIIβ genes silencing. Control: non-transduced cells. S: cells transduced with scrambled shRNA. (**C**,**F**) Silencing the NMT1 or CKIIα genes blocked Tat.AG-induced increase in CAT enzyme levels (**C**) and luciferase activity (**F**), but had no effect on Tat.B-induced viral transactivation. Silencing the NMT2 or CKIIβ genes had no effect on Tat.AG or Tat.B-induced viral transactivation. *P < 0.05, **P < 0.01, ^#^P < 0.0001, compared to cells treated with similar concentrations of scrambled (S) shRNA and Tat proteins. Errors bars represent SD.

Our previous analyses of Tat sequences from over 100 human plasma samples showed conserved post-translational modifications (PTMs) in Tat functional domains, including N-myristoylation and CKII domains, and demonstrated subtype-based differences in these Tat PTMs^12^. To determine whether these subtype-based differences in Tat PTMs can influence its function, we silenced two NMT (NMT1 and NMT2) and two CKII (CKIIα and CKIIβ) genes in U38 (Fig. 7A–C) and TZM-bl (Fig. 7D–F) cells, and assessed Tat-induced HIV-1 transactivation. Silencing of NMT1, NMT2, CKIIα and CKIIβ genes was confirmed by reverse-transcription (RT)-PCR (Fig. 7A,D) and Western blots (Fig. 7B,E); 5 × 10^4^ shRNA infectious units of lentiviral particles induced maximal gene silencing without cellular toxicity (Fig. 7A,B,D,E). Both Tat.AG and Tat.B (10–1000 ng/ml) significantly increased LTR transcription in U38 (Fig. 7C) and TZM-bl (Fig. 7F) cells, with Tat.AG inducing higher LTR transcriptional activity compared to Tat.B. Silencing the NMT1 gene blocked Tat.AG-induced LTR transcription: reduced Tat.AG-induced LTR transcription in U38 cells by 62–85% (Fig. 7C) and in TZM-bl cells by 33–60.5% (Fig. 7F) (P < 0.0001), but had no effect on Tat.B-induced LTR transcription (Fig. 7C,F). Silencing the CKIIα gene also blocked Tat.AG-induced LTR transcription: reduced Tat.AG-induced LTR transcription in U38 cells by 74–90% (Fig. 7C) and in TZM-bl cells by 52.7–64.8% (Fig. 7F) (P < 0.0001), but had no effect on Tat.B-induced LTR transcription (Fig. 7C,F). Silencing the NMT2 or CKIIβ genes had no effect on Tat.AG or Tat.B -induced LTR transcription (Fig. 7C,F).

### NMT1 and CKIIα mediate HIV-1 CRF02_AG, but not clade-B, replication in human macrophages

**Figure 8:**
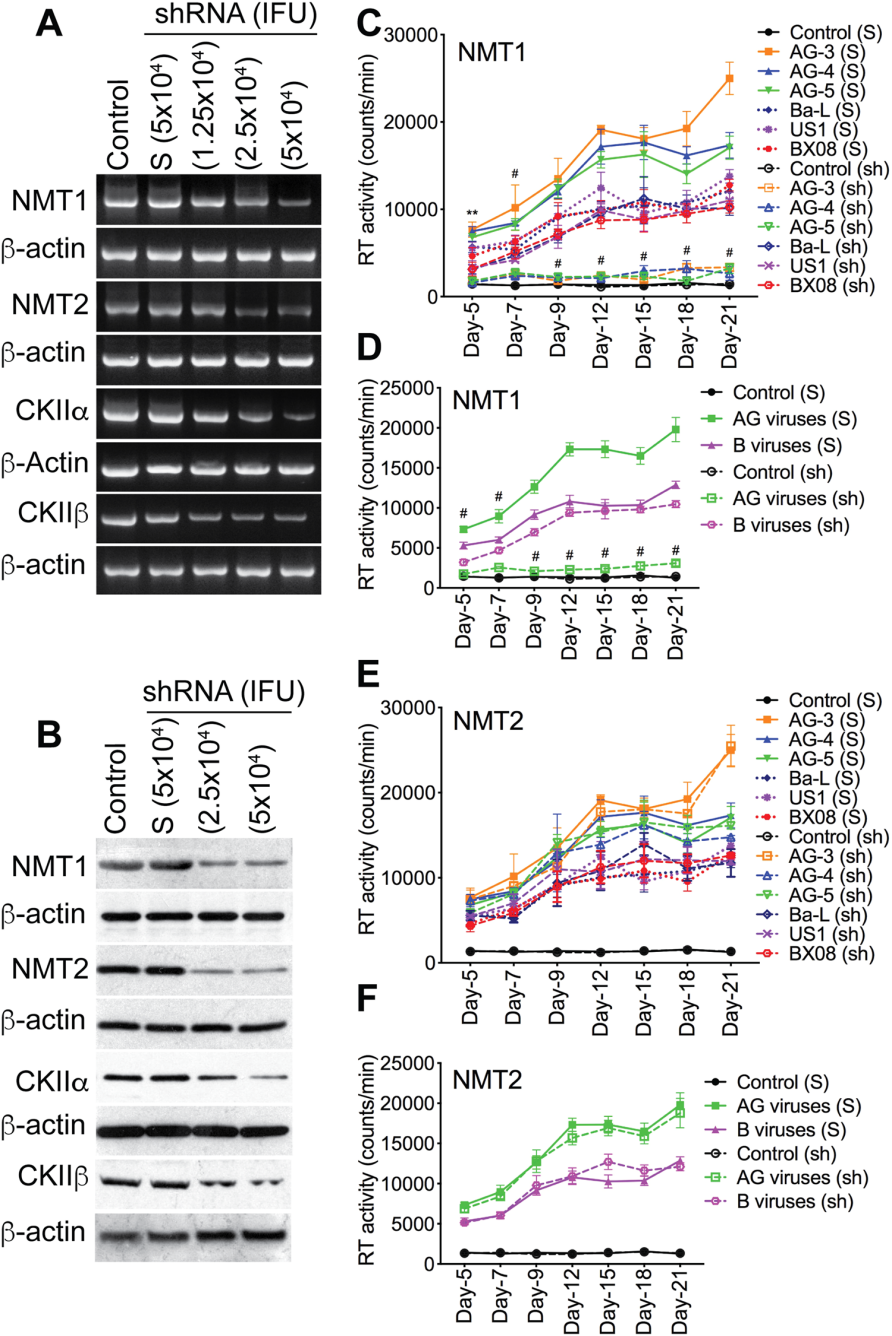
Silencing the NMT1 gene blocked the infection of human MDM by HIV-1 CRF02_AG isolates, but had no effect on clade-B HIV-1 infection. Silencing of NMT1, NMT2, CKIIα and CKIIβ genes in primary human MDM was confirmed by RT-PCR (**A**) and Western blot analyses (**B**). For panels **A** and **B**, controls are non-transduced MDM. “S” are MDM transduced with scrambled shRNA. Following NMT1 and NMT2 gene silencing, MDM were infected with CRF02_AG or clade-B HIV-1 isolates (3 different isolates for each subtype, each isolate tested in triplicate) and RT activity measured from day-5 to day-21 p.i. (**C**,**D**) RT activity in MDM with NMT1 gene silenced. (**E**,**F**) RT activity in MDM with NMT2 gene silenced. For panels **C**–**F**, controls are non-HIV infected (mock-infected) MDM, “S” are MDM transduced with scrambled shRNA; “sh” are MDM transduced with NMT1 or NMT2 shRNA. **P < 0.01; ^#^P < 0.0001, compared to infected MDM treated with similar concentrations of scrambled shRNA (S).

**Figure 9:**
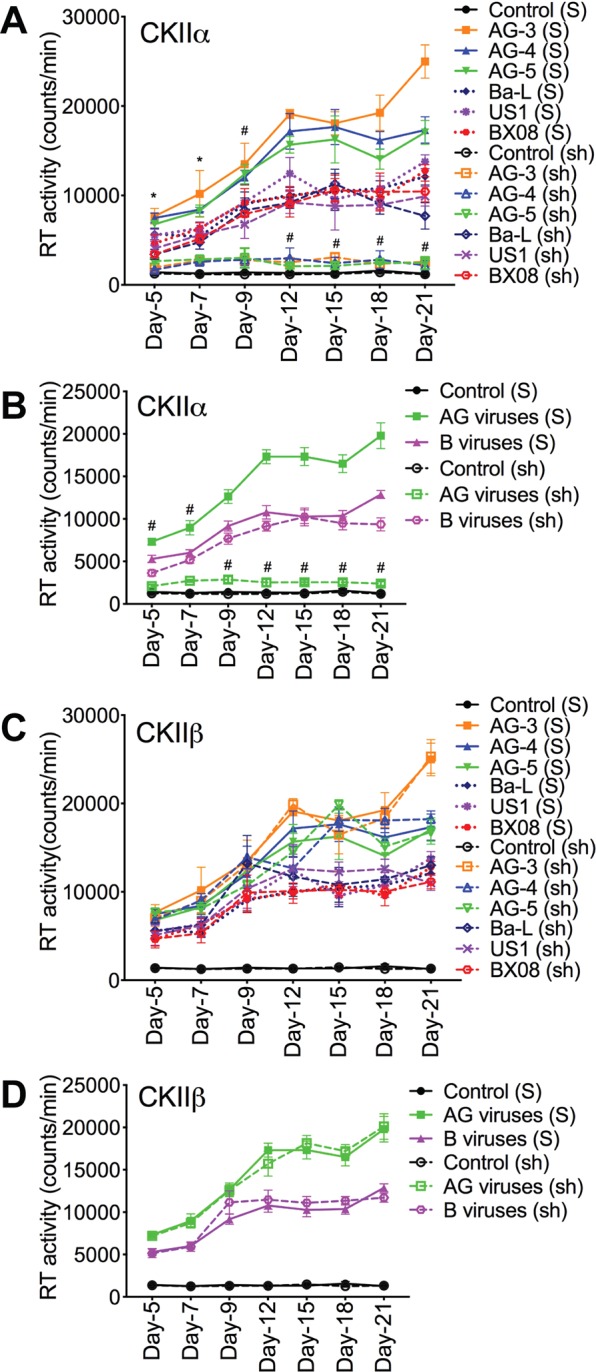
Silencing the CKIIα gene blocked the infection of human MDM by HIV-1 CRF02_AG isolates, but had no effect on clade-B HIV-1 infection. (**A**–**D**) Following silencing of CKIIα and CKIIβ genes, MDM were infected with CRF02_AG or clade-B HIV-1 isolates (3 different isolates for each subtype, each isolate tested in triplicate) and RT activity measured from day-5 to day-21 p.i. (**A**,**B**) RT activity in MDM with CKIIα gene silenced. (**C**,**D**) RT activity in MDM with CKIIβ gene silenced. Controls are non-HIV infected (mock-infected) MDM, “S” are MDM transduced with scrambled shRNA; “sh” are MDM transduced with CKIIα or CKIIβ shRNA. *P < 0.05, ^#^P < 0.0001, compared to infected MDM transduced with similar concentrations of scrambled shRNA (S).

To validate our data and determine whether CKII and/or protein myristoylation are involved in HIV-1 replication and whether there are subtype differences, we assessed the effects of NMT1, NMT2, CKIIα, and CKIIβ on HIV-1 CRF02_AG and clade-B replication in primary human macrophages. Silencing of NMT1, NMT2, CKIIα, and CKIIβ genes in MDM was confirmed by RT-PCR (Fig. 8A) and Western blot (Fig. 8B); 5 × 10^4^ infectious units of each shRNA induced maximal gene silencing without causing cellular toxicity. From day-5 to day-21 p.i., RT activity in MDM infected with AG viruses was consistently higher than RT activity in MDM infected with clade-B viruses (P < 0.0001, Figs 8 and 9). Silencing NMT1 blocked MDM infection by AG isolates but had no effect on infection by clade-B isolates (Fig. 8C,D). Compared to scrambled shRNA, NMT1 shRNA reduced HIV-1 CRF02_AG replication in MDM by 71.4–86.7% (P < 0.0001, Fig. 8C,D) but had no effect on the replication of clade-B viruses. From day-5 to day-21 p.i., the replication of clade-B viruses in MDM with silenced NMT1 gene was 1.8–4.1-fold higher than the replication of AG viruses in MDM with silenced NMT1 gene (P < 0.0001, Fig. 8C,D). Silencing the NMT2 gene had no effect on the replication of AG or clade-B viruses (Fig. 8E,F).

Additional experiments also confirmed the involvement of CKIIα in the replication of HIV-1 CRF02_AG, but not clade-B viruses. Silencing the CKIIα gene blocked MDM infection by HIV-1 CRF02_AG isolates, but had no effect on infection by clade-B isolates (Fig. 9A,B). Compared to scrambled shRNA, CKIIα shRNA reduced HIV-1 CRF02_AG replication in MDM by 71–87.8% (P < 0.0001, Fig. 9A,B) but had no effect on the replication of clade-B viruses. From day-5 to day-21 p.i., the replication of clade-B viruses in MDM with silenced CKIIα gene was up to 4-fold higher than the replication of AG viruses in MDM with silenced CKIIα gene (P < 0.0001, Fig. 9A,B). Silencing the CKIIβ gene had no effect on the replication of AG or clade-B viruses (Fig. 9C,D).

### Tat.AG and HIV-1 CRF02_AG, but not Tat.B or clade-B viruses, induced NMT1 expression and secretion in human monocytes and MDM

**Figure 10:**
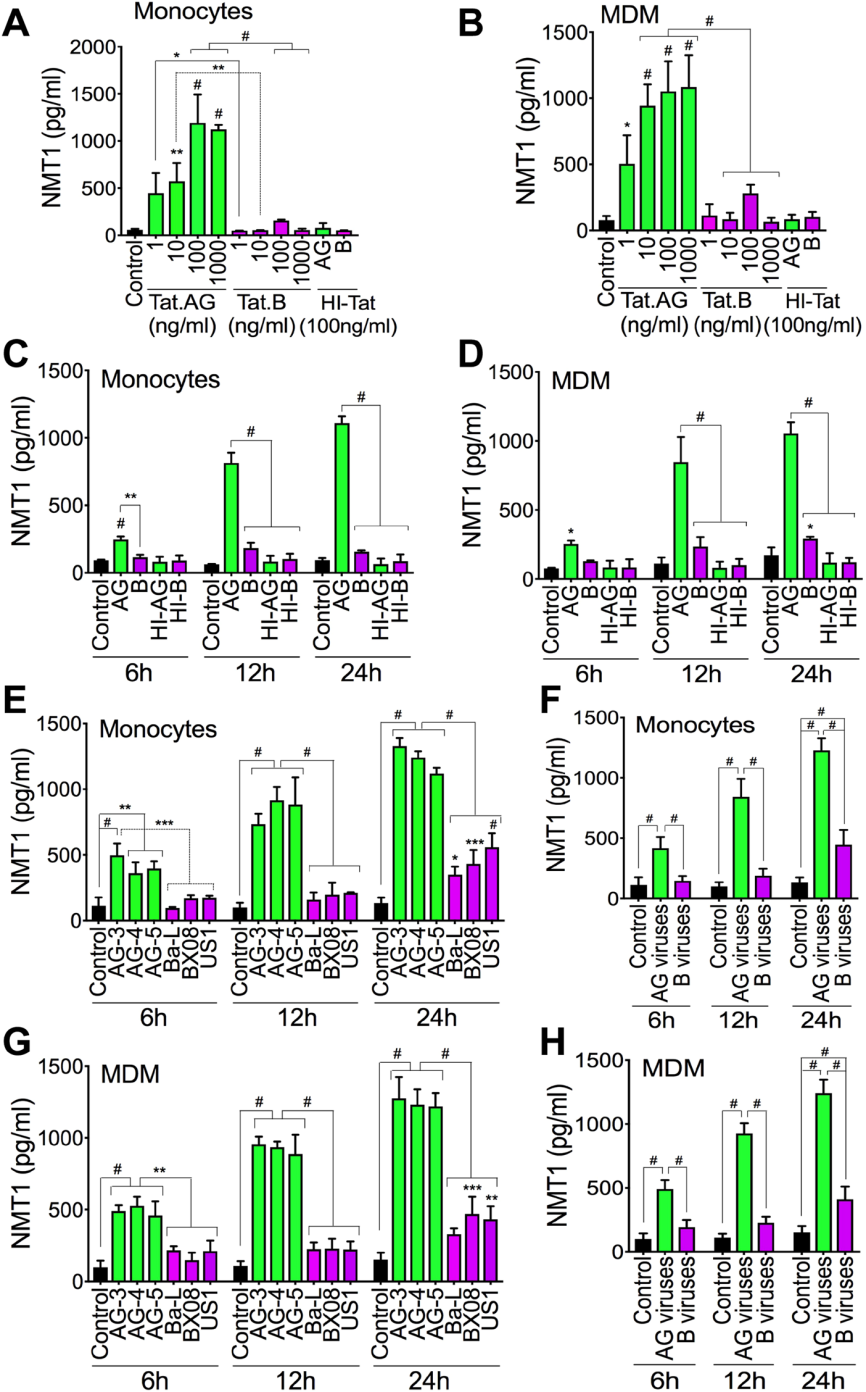
Tat.AG and HIV-1 CRF02_AG, but not Tat.B or clade-B viruses, induced NMT1 expression in primary human monocytes and MDM. (**A**–**D**) Exposure of human monocytes (**A**,**C**) and MDM (**B**,**D**) to Tat.AG induced a dose-dependent (**A**,**B**) and time-dependent (**C**,**D**) increase in NMT1 expression and secretion. (**A**,**B**) cells exposed to Tat proteins for 24 h; (**C**,**D**) Tat proteins used at 100 ng/ml. (**E**–**H**) Exposure of human monocytes (**E**,**F**) and MDM (**G**,**H**) to HIV-1 CRF02_AG isolates induced a time-dependent increase in NMT1 expression and secretion. *P < 0.05, **P < 0.01, ***P < 0.001, ^#^P < 0.0001. P-values are in comparison to HI-Tat or Tat.B (**A**–**D**), or clade-B viruses (**E**–**H**).

Compared to cells exposed to HI-Tat, Tat.AG increased NMT1 expression in monocytes by 5.5–14.88-fold (Fig. 10A) and in MDM by 6–13-fold (Fig. 10B). Tat.B did not induce much NMT1 in monocytes or MDM. NMT1 levels in monocytes and MDM exposed to Tat.AG were respectively 7.6–19.4-fold (Fig. 10A) and 3.75–16.4-fold (Fig. 10B) higher than NMT1 levels in monocytes and MDM treated with similar concentrations of Tat.B. Time-course experiments confirmed these findings; whereas Tat.B treatment of monocytes or MDM for 6–24 hours(h) did not induce NMT1, Tat.AG time-dependently increased NMT1 expression and secretion: treatment with Tat.AG for 6 h, 12 h, and 24 h increased NMT1 expression respectively by 3-fold, 9.74-fold, and 17.09-fold in monocytes (Fig. 10C) and by 3–10.4-fold in MDM (Fig. 10D), compared to cells exposed to similar concentrations of HI-Tat. Tat.AG also significantly increased NMT1 expression in U38 cells compared to Tat.B (data not shown).

CRF02_AG viruses time-dependently increased NMT1 expression and secretion in monocytes and MDM. Exposure of human monocytes and MDM to AG viruses for 6–24 h increased NMT1 expression respectively by 3.7–9.2-fold (Fig. 10E,F) and by 4.97–8.5-fold (Fig. 10G,H); whereas exposure of monocytes and MDM to clade-B viruses at similar multiplicity of infection (MOI) increased NMT1 levels respectively by 1.3–3.35-fold (Fig. 10E,F) and by 1.94–2.7-fold (Fig. 10G,H). Overall NMT1 levels were 2.75–4.45-fold higher in monocytes (Fig. 10E,F) and 2.56–4.1-fold higher in MDM (Fig. 10G,H) exposed to AG viruses compared respectively to NMT1 levels in monocytes and MDM exposed to clade-B viruses.

### Tat.AG and HIV-1 CRF02_AG, but not Tat.B or clade-B viruses, induce CKIIα expression in human monocytes and MDM

**Figure 11:**
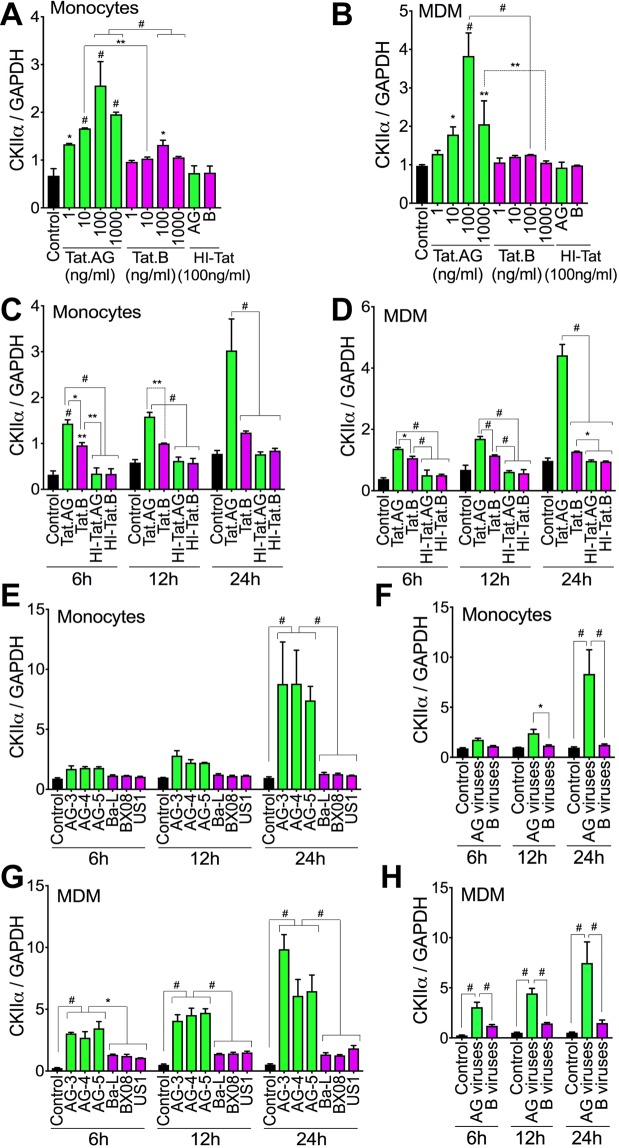
Tat.AG and HIV-1 CRF02_AG, but not Tat.B or clade-B viruses, induced CKIIα expression in primary human monocytes and MDM. (**A**–**D**) Exposure of human monocytes (**A**,**C**) and MDM (**B**,**D**) to Tat.AG induced a dose-dependent (**A**,**B**) and time-dependent (**C**,**D**) increase in CKIIα expression. (**A**,**B**) cells exposed to Tat proteins for 24 h; (**C**,**D**) Tat proteins used at 100 ng/ml. (**E**–**H**) Exposure of human monocytes (**E**,**F**) and MDM (**G**,**H**) to HIV-1 CRF02_AG isolates induced a time-dependent increase in CKIIα expression. *P < 0.05, **P < 0.01, ^#^P < 0.0001. P-values are in comparison to HI-Tat or Tat.B (**A**–**D**), or clade-B viruses (**E**–**H**).

Compared to cells exposed to HI-Tat, Tat.AG increased CKIIα expression in monocytes by 1.8–3.5-fold (Fig. 11A) and in MDM by 1.4–4-fold (Fig. 11B). Tat.B did not significantly increase CKIIα expression in monocytes or MDM (Fig. 11A,B). CKIIα levels in monocytes and MDM exposed to Tat.AG were respectively 1.4–1.94-fold (Fig. 11A) and 1.2–3-fold (Fig. 11B) higher than CKIIα levels in monocytes and MDM treated with similar concentrations of Tat.B. Treatment of monocytes and MDM with Tat.AG for 6–24 h increased CKIIα expression respectively by 2.5–4.2-fold (Fig. 11C) and by 2.7–4.5-fold (Fig. 11D), compared to cells exposed to similar concentrations of HI-Tat proteins. Tat.AG also significantly increased CKIIα in U38 cells compared to Tat.B (data not shown).

HIV-1 CRF02_AG time-dependently increased CKIIα expression in monocytes and MDM. Exposure of human monocytes and MDM to AG viruses for 6–24 h increased CKIIα expression respectively by 1.95–8.7-fold (Fig. 11E,F) and by 2.8–5-fold (Fig. 11G,H); whereas exposure of monocytes and MDM to similar MOI of clade-B viruses did not significantly increase CKIIα levels (Fig. 11E–H). Overall CKIIα levels in monocytes and MDM exposed to AG viruses were respectively 1.57–6.67-fold (Fig. 11E,F) and 2.5–5-fold (Fig. 11G,H) higher than CKIIα levels in monocytes and MDM exposed to similar MOI of clade-B viruses.

## Discussion

The epidemiology of HIV/AIDS is characterized by distinct and differential geographic subtypes distribution, with HIV-1 CRF02_AG prevalent throughout West and Central Africa^5–7^. This differential geographic distribution of HIV clades is likely associated with differences in viral fitness, transmission efficiency, replication capacity, and viral adaptation in a given environment. In the present study, we use cell lines, primary HIV-1 isolates, primary human PBMC, MDM, and *in-vivo* HIV/AIDS animal models to demonstrate significantly increased replication of HIV-1 CRF02_AG compared to clade-B isolates. *In-vivo* and *ex-vivo* data also showed higher plasma viremia and significantly higher viral load in the heart, kidney, liver, spleen, and lung tissues of AG-infected animals compared to animals infected with clade-B viruses; indicating a generalized increased replication of AG isolates throughout animals’ blood and tissues.

Previous studies in activated T-cells^25^ and PBMC^24^ also showed increased *ex-vivo* replicative fitness of CRF02_AG isolates compared to its parental HIV-1 subtypes A and G^24,25^ and independently of patient’s CD4 counts or co-receptor use^25^. It is therefore likely that the increased replication capacity of HIV-1 CRF02_AG is responsible for its predominance throughout West and Central Africa. The associated mechanisms are not known; elucidating the mechanisms responsible for this increased replication of HIV-1 CRF02_AG strains is critical to understanding viral epidemiology, including its transmission and propagation in Sub-Saharan Africa. In the present study, we show that in addition to the increased replication of CRF02_AG isolates in primary human cells and *in-vivo*, these AG isolates showed significantly higher LTR transactivation compared to clade-B viruses. Tat proteins are expressed early in the HIV life cycle and transactivate the LTR to induce the expression of HIV genes and viral replication. Our data showing significantly higher transactivation of HIV-1 LTR with Tat.AG, compared to Tat.B, further confirm that viral genotype influence the magnitude of LTR transactivation and viral replication.

Increased HIV-1 transactivation and viral replication are associated with histone acetylation and increased HAT activity; whereas decreased HIV-1 transactivation, lower viral replication, and viral latency are associated with histone deacetylation and increased histone deacetylase activity^29–31^. Our current study shows that increased transactivation and replication of CRF02_AG isolates correlated with increased HAT activity and acetylation of histone H3 and H4, with higher levels of acetylated H3 and H4 observed with Tat.AG, compared to Tat.B. These data suggest that the increased replicative capacity of CRF02_AG isolates involves epigenetic modifications, especially acetylation of histones H3 and H4 in the Nu-0 and Nu-1 of the viral promoter.

PTMs of proteins modulate their structure and function, including signaling and interactions with other molecules and co-factors. Our previous analyses of Tat sequences in plasma from over 100 HIV-1-infected subjects showed the presence of conserved PTMs in Tat functional domains, including CKII and N-myristoylation domains^12^, with CRF02_AG Tat sequences having two N-myristoylation domains (in the Core and N-terminal regions) and a CKII domain (in the glutamine-rich region) compared to Tat sequences from other HIV-1 subtypes^12^. Therefore, we investigated whether N-myristoylation and/or CKII play a role in the differential transactivation and viral replication observed. Analyses using two different cell types containing integrated silent copies of the HIV-1 promoter showed increased viral transactivation with Tat.AG compared to Tat.B, and further showed that silencing the NMT1 or CKIIα genes blocked Tat.AG-induced HIV transactivation but had no effect on Tat.B-induced viral transactivation; whereas silencing the NMT2 or CKIIβ genes had no effect on Tat.AG- or Tat.B-induced viral transactivation. Significantly, we showed that silencing the NMT1 or CKIIα genes blocked human macrophage infection by HIV-1 CRF02_AG isolates but had no effect on infection by clade-B isolates, and silencing the NMT2 or CKIIβ genes had no effect on macrophage infection by both viral subtypes.

CKII is a serine/threonine selective protein kinase that preferentially phosphorylate serine and threonine residues^32,33^, and has been shown to play a role in HIV infection and HIV/AIDS pathogenesis^34^. CKII-mediated phosphorylation of Rev, Vpu, and protease at serine residues facilitates viral infection, syncytia formation, and disease progression^34–38^. CKII-mediated phosphorylation of Rev serine residues also influences Vpu-CD4 interactions^39,40^; and mutations in the Vpu CKII site significantly altered its biological activity^41^. Our current data shows that CKIIα differentially mediates Tat transactivation and HIV replication based on viral genotype, with AG viruses more susceptible to CKIIα-mediated viral transactivation and replication compared to clade-B viruses. In the NFκB pathway, IκB phosphorylation and degradation results in NFκB translocation to the nucleus where it activates target genes^42–44^. HIV-1 has NFκB binding sites and this pathway play a major role in viral transactivation and replication^45–47^. CKII associates with IκBα, is required for basal and HIV-1-induced IκBα degradation^48^, and CKIIα phosphorylates IκBα at serine and threonine residues to induce IκBα degradation and NFκB signaling^48^. Our current study shows that this CKIIα subunit modulates the transactivation and replication of CR02_AG HIV-1 isolates, whereas the CKIIβ subunit has no effect, suggesting that targeting this alpha subunit could help abrogate the replication and expansion of AG isolates.

Myristoylation is a lipidic modification whereby a myristoyl group is covalently attached to an N-terminal glycine residue^49^. Myristoylation can occur co-translationally or post-translationally, and is catalyzed by NMT using myristoyl-coenzyme-A as substrate^49^. Higher eukaryotes have two NMT isozymes (NMT1 and NMT2) encoded by two genes that share about 70% similarity^50^. Myristoylation increases protein-protein interactions, guides protein sub-cellular localization, intra-cellular trafficking, signaling, and immune responses^49,51^. Myristoylation has been shown to play an important role in cell viability, cell survival, innate and adaptive immune response, and HIV infection^49,52,53^. Myristoylation is critical for HIV-1 Nef function and is involved in Nef-CD4 interactions^54,55^. Myristoylation also play a major role in Gag function and disrupting Gag myristoylation block HIV-1 replication, formation and release of new virions^56–58^. In fact, NMT targets Gag on the cell membrane^57,58^ and this Gag myristoylation enables protein-protein and protein-lipid interactions, resulting in the recruitment of Gag, viral RNA and proteins to the host’ cellular membrane and budding of new virions^56,57,59^. It has also been shown that NMT1 is the predominant isoform involved in Nef-CD4 interactions^60^ and HIV-1 production^61^. Our current study shows that NMT1 modulates the transactivation and replication of CR02_AG HIV-1 isolates, suggesting that targeting NMT1 could help abrogate the transactivation and replication of AG isolates.

The potential implications of this higher replicative fitness of HIV-1 CRF02_AG are significant, because higher AG replicative fitness can influence viral transmission and HIV/AIDS epidemiology. In fact, it has been shown that increased AG replicative fitness also influence its transmission in humans. Monitoring of at-risk seronegative women in West-Africa showed that among subjects who became seropositive, those infected with HIV-1 CRF02_AG had significantly higher viral loads during the early stages of infection compared to those infected with non-AG HIV-1^62^. Studies of HIV-infected humans in Sub-Saharan Africa showed that higher HIV replication capacity was associated with differential inflammatory states and increased T-cells activation^63^, increased risk of HIV transmission to other humans, including increased risk of mother-to-child transmission^64^, and that there was a positive correlation between high *ex-vivo* HIV replicative capacity and the magnitude of viral burden *in-vivo*^63^.

Paradoxically, higher viral loads in HIV-1 CRF02_AG-infected animals did not correlate with increased immunosuppression or wasting syndrome, as animals infected with HIV-1 subtype-B showed significantly more weight loss, lower hCD4+ T-cells and lower hCD4/hCD8 ratios. This suggest that factors other than viremia may be playing a role in HIV-associated immunosuppression and weight loss. Inflammation could be one such factor, as we previously showed differential inflammatory effects of Tat based on viral subtypes, including significant increase in inflammatory cytokines, chemokines, and matrix metalloproteinases, as well as increase and activation of complement factors in primary human brain endothelial cells exposed to Tat.B, compared to cells exposed to Tat.AG^19,20^. Our subsequent studies will investigate the association between inflammation, immunosuppression, and wasting syndrome between these viral subtypes.

Taken together, our current data showed significantly higher replication of HIV-1 CRF02_AG *in-vitro* and *in-vivo*, compared to HIV-1 subtype-B, and provide insights into the molecular mechanisms by which epigenetics, CKII-alpha, and NMT1 modulates this increased replication of CRF02_AG viruses. The fact that silencing the NMT1 and CKIIα genes blocked the infection of human cells by HIV-1 CRF02_AG but not clade-B viruses, suggests that the additional N-myristoylation and CKII domains present in Tat.AG sequences are functional and directly modulate the replication of CRF02_AG viruses. Such genetic differences should be considered when designing strategies to curb HIV replication. Our current studies have implications for viral eradication strategies and HIV/AIDS epidemiology, including the transmission, adaptation, and predominance of this recombinant virus across West-Central Africa.

## Materials and Methods

### Human monocytes, MDM, PBMC, PBL cultures

Monocytes, PBMC, and PBL were obtained by countercurrent centrifugal elutriation of leukopheresis packs from HIV-1, -2 and hepatitis-B seronegative donors (obtained from the Omaha American Red Cross), and cultured as previously described^65,66^. To obtain MDM, freshly elutriated monocytes (2 million cells per well in 6-well plates) were differentiated into MDM by culture for 7 days in Dulbecco’s Modified Eagle’s Medium (DMEM, Sigma, St. Louis, MO) supplemented with 10% heat-inactivated pooled human serum, 1% glutamine, 50 µg/ml gentamicin, 10 µg/ml ciprofloxacin (Sigma), 1000 U/ml highly purified recombinant human macrophage colony stimulating factor (hMCSF), and cultured as we previously described^65,66^. All reagents were prescreened for endotoxin (<10 pg/ml, Associates of Cape Cod, Woods Hole, MA) and mycoplasma contamination (Gen-probe II, Gen-probe, San Diego, CA). For cells from each donor, each experimental condition was performed in triplicate, and experiments were repeated using cells from two other donors (minimum of 3 different donors).

### HIV-1 infection

HIV-1 clade-B and CRF-02_AG were used. The clade-B isolates [and their GenBank accession numbers] were: MN (84US_MNp [M17449]), Ba-L (85US_Ba-L [AY713409]), US1 (91US_1 [AY173952]), US4 (91US_4 [AY173955]), US33393 (94US_33931N [AY713410]), US873 (90US_873 [AY713412]), BX08 (92FR_BX08 [AY713411])^67^, and the laboratory-adapted HIV-1_ADA_^47,65^. The primary HIV-1 CRF02_AG isolates were: AG-1 (CMNYU2103), AG-2 (02_CM0014BBY [AY371126]), AG-3 (02CM_1970LE [AY371128]), AG-4 (01CM_1475MV [AY371138]), AG-5 (01CMNYU5466 [AY359796])^67,68^.

Primary HIV-1 strains were propagated in phytohemagglutinin-stimulated human PBMC and titrated as previously described^47,69^. For infections, cells were cultured in media containing HIV-1 for 4 h (each viral strain at MOI of 0.01; each experimental condition tested in triplicate), washed 3 times with serum-free media and cultured for up to 21 days, with culture media changed every 2 or 3 days. RT activity was quantified as we previously described^47^.

### Tat.AG and Tat.B

Recombinant Tat proteins from a subtype-B HIV-1 isolate (Tat.B) (amino acids 1 to 86; accession number: P69697) were purchased from Diatheva (Viale Piceno, Fano, Italy). Recombinant Tat proteins from HIV-1 CRF02_AG (Tat.AG) (amino acids 1 to 86; accession number: AY371128) were made by Diatheva under a custom-order agreement with our laboratory, using similar procedures as for Tat.B^19,20^. For controls, HIV-1 Tat proteins were heat-inactivated at 100 °C for 30 minutes (min), cooled down and centrifuged at 78 g for 5 min to recover all of the solution. Protein aliquots were stored at −80 °C.

### U38 and TZM-bl cells

Monocytic U38 cells are derivative of U937 cells that contains stably integrated, silent copies of the HIV-1 LTR linked to the chloramphenicol acetyltransferase (CAT) gene. TZM-bl cells stably express large amounts of CD4 and CCR5 and contain integrated copies of the luciferase and β-galactosidase genes under the control of the HIV-1 promoter. U38 and TZM-bl cells were obtained from the NIH AIDS Research and Reference Program and cultured as previously described^47^.

### Histone acetyltransferase (HAT) assay

Following infection and cellular treatment, nuclear extracts were prepared using the Epigentek nuclear extraction kit (Epigentek, Farmingdale, NY) and total HAT activity in nuclear extracts (20 μg protein) quantified using the EpiQuik^TM^ HAT Activity /Inhibition Assay Kit (Epigentek) as previously described^47^.

### Chromatin immunoprecipitation (ChIP) analysis

ChIP assays were performed using the ChromaFlash^TM^ Chromatin Extraction Kit and ChromaFlash^TM^ One-Step ChIP kit (Epigentek) as we previously described^47^. Briefly, following treatment, cells pellets were resuspended in ice-cold phosphate-buffered saline (PBS) containing 0.5 mM phenylmethylsulphonyl fluoride, and cellular chromatin was sheared using a closed system ultrasonic cell disruptor (Microson^TM^, Qsonica LLC, Newtown, CT). Sheared samples were centrifuged (17,606 g, 10 min at 4 °C) and chromatin aliquots (supernatants) immunoprecipitated with acetylated H3 and H4 antibodies (Active Motif, Carlsbad, CA) using ChromaFlash^TM^ One-Step ChIP kit (Epigentek), per manufacturer’s protocol. Controls included aliquots (10%) of each sheared chromatin (“input” DNA control) and chromatin samples immunoprecipitated with isotype-matched control IgG. Immunoprecipitated samples were amplified by PCR using LTR-specific primers. PCR cycle was as follows: 95 °C, 3 min denaturation; followed by 30 cycles of 95 °C, 20 sec, 55 °C, 20 sec, and 72 °C, 8 sec; 72 °C, 1 min, and hold at 10 °C. Amplified samples were analyzed by agarose gel electrophoresis using 2.5% agarose, and images captured using the G-BOX gel-doc system (Syngene).

### Chloramphenicol acetyltransferase (CAT) and iuciferase assays

Following treatment, U38 cells were washed with PBS, lysed using the lysis buffer of the CAT-ELISA kit (Roche Diagnostics Indianapolis, IN), and protein levels quantified using the bicinchoninic acid assay (BCA) assay^65^. The amount of CAT enzyme in each sample (150 μg protein) was quantified as we previously described^47^, using the CAT-ELISA kit and standards (Roche) per manufacturer’s protocol.

The luciferase reporter assay was performed as previously described^47^ using the Luciferase Assay System (Promega, Madison, WI), per manufacturer’s protocol. Briefly, following treatment, cells were washed with PBS, and lysed using the Luciferase Assay Reporter System lysis reagent. Cell lysates (20 μl containing 50 μg proteins) were mixed with 100 μl Luciferase Assay Reagent and luciferase activity was measured using SpectraMax-M5 microplate reader (Molecular Devices, Sunnyvale, CA).

### RNA extraction

Total RNA was extracted from treated cells or animal tissues using the Trizol reagent (Life Technologies-Ambion, Austin, TX), per manufacturer’s protocol. The RNA was further cleaned using Total RNA cleanup kit (Qiagen, Valencia, CA). RNA yield and quality were checked using a NanoDrop spectrophotometer (NanoDrop Technologies, Wilmington, DE) and for all samples, absorbance ratio of 260/280 was ≥2.

### Real-time PCR

Each experimental condition was tested in triplicate and for each replicate sample, cDNA was generated from 1 μg RNA in a 20 μl reaction volume, using the Verso cDNA kit (Thermo Fisher) per manufacturer’s protocol. Reverse transcription and qPCR were performed as previously described^47,70^, using LightCycler® 480 II (Roche) Real-Time PCR System. Human CD45 and CD4 qPCR was used as internal controls to normalize gene expression. These controls were used at the same primer-probe ratio as the target genes (900 nM of each primer and 250 nM TaqMan MGB probe).

ACH-2 cells were used for normalization based on hCD45+ cells levels in tissues. ACH-2, an HIV-1 latent T-cell clone containing one integrated copy of proviral DNA per cell, was obtained from the NIH AIDS Reagent Program. Standard curves from ACH-2 qPCR were used to quantify HIV-1 LTR, pol, tat, and gag copy numbers in each sample, and results were further normalized to levels of human CD45 cells in each tissue sample. For normalization based on hCD4+ cells levels in tissues, qPCR for hCD4 and HIV-1 LTR, pol, tat, gag was performed for each tissue sample and gene expression levels were quantified using the cycle threshold (Δ*C*_T_) method as described in the software user manual of LightCycler® 480 II Real-Time PCR System. Each viral gene expression was normalized to the sample hCD4. All PCR reagents, primers, and probes were from Applied Biosystems, and primers’ IDs were as follows: LTR (AIWR3QG), pol (AIY9Z2W), tat (AIX01W0), gag (AIo1X84), CD45 (Hs04189704), and CD4 (Hs01058407).

### Hu-PBL-NSG mice model

Four-week old NOD/*scid*–IL-2Rγ_c_^*null*^ (NSG) mice were purchased from the Jackson Laboratory (Bar Harbor, ME), maintained in sterile microisolator cages under pathogen-free conditions in accordance with UNMC and NIH ethical guidelines for care of laboratory animals, and bred at the UNMC animal facility to expand the colony. This study was performed under a protocol approved by the UNMC Institutional Animal Care and Use Committee. Mice, 4 to 6 weeks old male, were engrafted by intra-peritoneal (i.p.) injection of human PBL (30 × 10^6^ cells/mouse). One week after PBL injection, levels of human CD45 cells in each mouse’s blood sample were quantified by FACS to confirm engraftment. Engrafted animals were randomly assigned into 7 groups (10–12 mice per group): a non-infected control group, 3 groups infected with 3 different HIV-1 CRF02_AG strains (AG-3, AG-4, AG-5), and 3 groups infected with 3 different HIV-1 clade-B strains (Ba-L, US1, BX08). For infection, a single dose of 10^4^ tissue culture infectious doses-50 (200 μl) of the corresponding HIV-1 strain was injected (i.p.) to animals. Controls were mock-infected by i.p. injection of PBS (200 μl). Animals’ blood samples were collected and analyzed at week-1, -2, -3 p.i. Animals were sacrificed at week-3 p.i. and tissues samples harvested and analyzed.

### FACS analysis

Human (hCD4+, hCD8+, hCD3+, and hCD45+) cells in animal’s blood were quantified by FACS, using the following anti-human antibodies: CD45-PE-Cy7, CD8-APC, CD4-FITC and CD3-Pacific blue (Biolegend, San Diego, CA). Briefly, blood (200 μl) collected in EDTA-tubes were centrifuged (543 g, 8 min), plasma collected and cryopreserved, cell pellets resuspended in 50–200 μl FACS buffer (PBS containing 2% fetal bovine serum) and transferred into 5 ml polypropylene round-bottom tubes (BD Falcon, Franklin Lakes, NJ). Antibody cocktail including CD45-PE-Cy7, CD8-APC, CD4-FITC and CD3-Pacific blue was added to each sample and the mixture incubated 1 h on ice. One ml red blood cells lysis buffer (Roche) was then added to each sample, followed by 5 min incubation at RT and centrifugation (377 g, 5 min). Cells pellets were washed 4 times using the FACS buffer, and resuspended in PBS containing 2% paraformaldehyde and analyzed using BD LSRII and FACSDiva 8.0.

### HIV-1 p24 ELISA

HIV-1 p24 antigen levels in each plasma sample (100 µl) were quantified using the Quantikine HIV-1 Gag p24 immunoassay kit (R&D systems, Minneapolis, MN) per manufacturer’s protocol; with optical density (OD) readings at 450 nm and wavelength corrections at 540 nm, using a SpectraMax-M5. Standard curves from HIV-1 Gag p24 antigen standards were used to quantify samples p24 antigen levels.

### N-myristoyltransferase-1 and CKIIα ELISA

Monocytes and MDM in 96-well plates (1 × 10^6^ cells/well) were exposed to Tat or HIV-1 for 6–24 h; culture supernatants were then used for NMT1 ELISA and cells used for CKIIα ELISA. NMT1 levels in supernatants (100 µl) were quantified using the human NMT1 ELISA Kit (Aviva Systems Biology, San Diego, CA) per manufacturer’s protocol. For CKIIα, cells were washed, fixed for 20 min in 4% (MDM) or 8% (monocytes) paraformaldehyde, quenched, and CKIIα levels quantified using the human CKIIα ELISA kit (Aviva), per manufacturer’s protocol. OD readings were at 450 nm with wavelength corrections at 540 nm. CKIIα analyses were performed using anti-CKIIα or anti-GAPDH antibody and each sample’s CKIIα OD reading was normalized to its’ GAPDH OD value. NMT1 standards were used to quantify samples’ NMT1 levels.

### Short hairpin RNA (shRNA) and gene silencing

Knockdown of NMT1, NMT2, CKIIα and CKIIβ genes was performed using transduction ready shRNA containing 3 target-specific constructs that encoded 19–25 nucleotide (plus hairpin) shRNA designed to knockdown gene expression: NMT1 (sc-61132-V), NMT2 (sc-61134-V), CKIIα (sc-29918-V), and CKIIβ (sc-29916-V) (Santa Cruz Biotechnology, Dallas, TX). Each experiment also included control shRNA lentiviral particles (sc-108080, Santa Cruz Biotechnology) containing an shRNA construct encoding a scrambled sequence that was not expected to degrade any known cellular mRNA. Lentiviral transductions were performed using Polybrene Reagent (Santa Cruz Biotechnology) per manufacturer’s protocol. Gene knockdown was monitored by Western blot and RT-PCR, using the following Santa Cruz primers: NMT1 (h)-PR (sc-61132-PR), NMT2 (h)-PR (sc-61134-PR), CKIIα′ (h)-PR (sc-38963-PR), CKIIβ (h)-PR (sc-29916-PR), and β-Actin (h)-PR (sc-108069-PR) per manufacturer’s instructions.

### Western blot analysis

Cells were lysed using the mammalian cell lysis buffer CelLytic M (Sigma); total protein quantified using the BCA assay, and 30 μg protein analyzed by sodium dodecyl sulfate-polyacrylamide gel electrophoresis as previously described^47,65^ using monoclonal antibodies to NMT1, NMT2, CKIIα and CKIIβ (Santa Cruz). *β*-actin antibody (Abcam, Cambridge, MA) was used to confirm equal loading.

### Statistical analyses

Data were analyzed by unpaired t-test (two-tailed) for two-group comparisons. For multiple groups comparisons, data were analyzed by one-way ANOVA followed by Tukey’s multiple-comparisons tests; or by two-way RM ANOVA followed by Bonferroni or Sidak’s multiple-comparisons tests. GraphPad Prism 7.0d (GraphPad Software, La Jolla, CA) was used for analyses and the threshold of significance level was 0.05. Data are presented as means ± standard deviation (SD) or standard error of the mean (SEM).

### Ethical approval

Animal studies were conducted using an approved IACUC protocol, in accordance with UNMC and NIH ethical guidelines for care of laboratory animals.

## Data Availability

All data associated with this manuscript are available upon request.
